# Neuro-Muscular Dentistry: the “diamond” concept of electro-stimulation potential for stomato-gnathic and oro-dental conditions

**DOI:** 10.1186/s13005-021-00257-3

**Published:** 2021-01-26

**Authors:** Catalina P. Sandoval-Munoz, Ziyad S. Haidar

**Affiliations:** 1 BioMAT’X (Laboratorio de Biomateriales, Farmacéuticos y Bioingeniería de Tejidos Cráneo Máxilo-Facial), Universidad de los Andes, Mons. Álvaro del Portillo 12.455 - Las Condes, Santiago, Chile; 2 Programa de Doctorado en BioMedicina, Facultad de Medicina, Universidad de los Andes, Mons. Álvaro del Portillo 12.455 - Las Condes, Santiago, Chile; 3 Centro de Investigación e Innovación Biomédica (CIIB), Universidad de los Andes, Mons. Álvaro del Portillo 12.455 - Las Condes, Santiago, Chile; 4 Facultad de Odontología, Universidad de los Andes, Mons. Álvaro del Portillo 12.455 - Las Condes, Santiago, Chile

**Keywords:** Electric stimulation therapy, Oropharyngeal dysphagia, Temporomandibular joint disorders, Dental care, Speech-language pathology

## Abstract

Oro-Pharyngeal Dysphagia - or simply dysphagia - is the difficulty (persistent) in swallowing/passing food and/or liquid from the mouth to the pharynx into the esophagus and finally the stomach; a deglutition disorder (a symptom, by definition, often due to neuro-degenerative/−muscular, drug-induced or localized structural pathologies such as head and neck tumors, lesions and associated surgical and/or radiation injuries) linked to severe consequences on Quality of Life (QoL), including malnutrition, dehydration, and even sudden death. Likewise, Temporo-Mandibular Jaw and Joint disorder(s) – or simply TMD – is a multifactorial etiological condition, regularly encountered in the dental office. Whether due to malocclusion, bruxism, stress and/or trauma, TMD destabilizes the whole cranio-mandibular system structurally and functionally, via affecting mastication, teeth, supporting structures, comfort and aesthetics, and thus, QoL, again. While several treatment regimens do exist for such conditions, some of which have been standardized for use over the years, most continue to lack proper evidence-based literature support. Hence, (1) caution is to be exercised; and (2) the need for alternative therapeutic strategies is amplified, subsequently, the door for innovation is wide open. Indeed, neuromuscular electrical stimulation or “NMES”, is perhaps a fine example. Herein, we present the interested oro-dental health care provider with an up-dated revision of this therapeutic modality, its potential benefits, risks and concerns, to best handle the dysphagic patient: an intra-disciplinary approach or strategy bridging contemporary dentistry with speech and language therapy; a rather obscure and un-discovered yet critical allied health profession*.* A pre-clinical and clinical prospectus on employing inventive NMES-based regimens and devices to manage TMD is also highlighted.

## Introduction

Our masticatory system, physiologically, governs a functional homeostasis between teeth, periodontium, masticatory muscles, temporo-mandibular jaw joints, and the surrounding cranio-facial neuromuscular and skeletal complex, thereby controlling jaw movement(s) [1]. Indeed, *occlusion is a foundation for dentistry*. Occlusal interferences, whether due to dentition fracture and/or pathological occlusal wear (commonly, erosion, attrition and abrasion), are often the pre-disposing factor in the development of Temporo-Mandibular Disorders or TMDs: a group of musculo-skeletal disorders affecting the form and/or function of the bi-lateral temporo-mandibular jaw joints, masticatory muscles, teeth, and supporting structures (surrounding facial nerves and muscles that control jaw movement), often presented as a cycle of pain and muscle spasms occurring when the triad of teeth, muscles and temporo-mandibular jaw joints lose their *harmonious* functional balance [2]. Hence, Neuro-Muscular Dentistry or NMD [3] has been defined as the three-dimensional (teeth, muscles and joints) science and art for incorporating or re-establishing a balanced and relaxed patho-physiology for anatomy, function and form/aesthetics – *an optimal relationship between the mandible and the skull*. Now, while several bi-directional associations have been correlated between dental occlusion, TMDs, the neuro-muscular system and body posture, primarily the cervical and scapular regions, it is well-established today that any alterations in mandibular muscles do result in deviations from the routine head posture, thereby directly linking any anatomical, neurological, biomechanical and/or pathological changes in the cervical spine and the cranio-maxillo-facial complex [3]. Yet, dentists tend to halt their investigation, diagnosis as well as treatment planning here, overlooking other possible associations, etiologies and consequences, some of which might be life-threatening. Indeed, predominantly the end-result of a number of pathologies (the most common being *stroke*), Oro-Pharyngeal Dysphagia or simply *dysphagia* (OPD) is a persistent swallowing symptom/disorder characterized by the inefficiency and dysfunction of one or more parts of the swallowing apparatus which begins with the mouth/oral cavity and includes the lips, tongue, jaws, pharynx, and the esophagus (and its upper and lower sphincters) [4]. OPD, most common in elderly/geriatric patients (although can affect all age groups yet risk increases with age and ageing - termed *presbyphagia*, if occurring naturally and is an age-related deterioration), also results from various neuro-muscular/−degenerative, drug-induced, and structural aetiologies; such as head trauma, Parkinson’s, Alzheimer’s/dementia, Multiple Sclerosis, Kearns-Sayre syndrome, head and neck tumours, and radiation injury [4, 5]. Consequently, OPD substantially impacts and reduces Quality of Life (QoL), increases the risk of medical complications and mortality, and poses a substantial cost to health-care systems (especially for stroke-related bounce-backs) [6]. This is especially true in light that OPD treatment, often following an endoscopy to try and determine the exact etiology, is diagnosis-specific and can be complicated by the co-presence of other diseases and disorders in the patient. Hence, no single medication or surgical corrective approach can be pin-pointed herein [7]. Further, alterations to dilution, swallowing difficulties and OPD cannot be prevented, can be life-threatening and can cause choking, and often lead to mal-nutrition (plus deficiencies in vitamins and minerals), weight loss, de-hydration, muscle breakdown, fatigue, as well as aspiration pneumonia (introduction of bacteria into the lungs), chronic lung disease(s) and death. Emotional impairment and socio-economic restrictions have been previously detected. Moreover, *dysarthria,* a disorder in the muscular control for speech (thereby, impaired communication), have been reported, often in patients already suffering from Parkinson’s [4–7]. Hence, in the older/geriatric population, patient’s survival/health, safety and QoL become a priority, requiring crucial, time-sensitive and often perplexing clinical decisions. An example is whether to recommend diet modification and the use of feeding tubes as a “compensatory” measure. Another interventional strategy can be considered “rehabilitative”, at the anatomical and biological levels, involving surgical procedures. Such critical decisions are made and executed by the team providing care to the patient after deliberate consultations and challenging discussions with the patient and family. Meanwhile, to improve symptoms caused by narrowing or scarring of the esophagus, pharmacological formulations targeting esophageal motility disorders that cause esophageal dysphagia specifically such as achalasia and diffuse esophageal spasms, to mention a few [8] do exist.

**Fig. 1:**
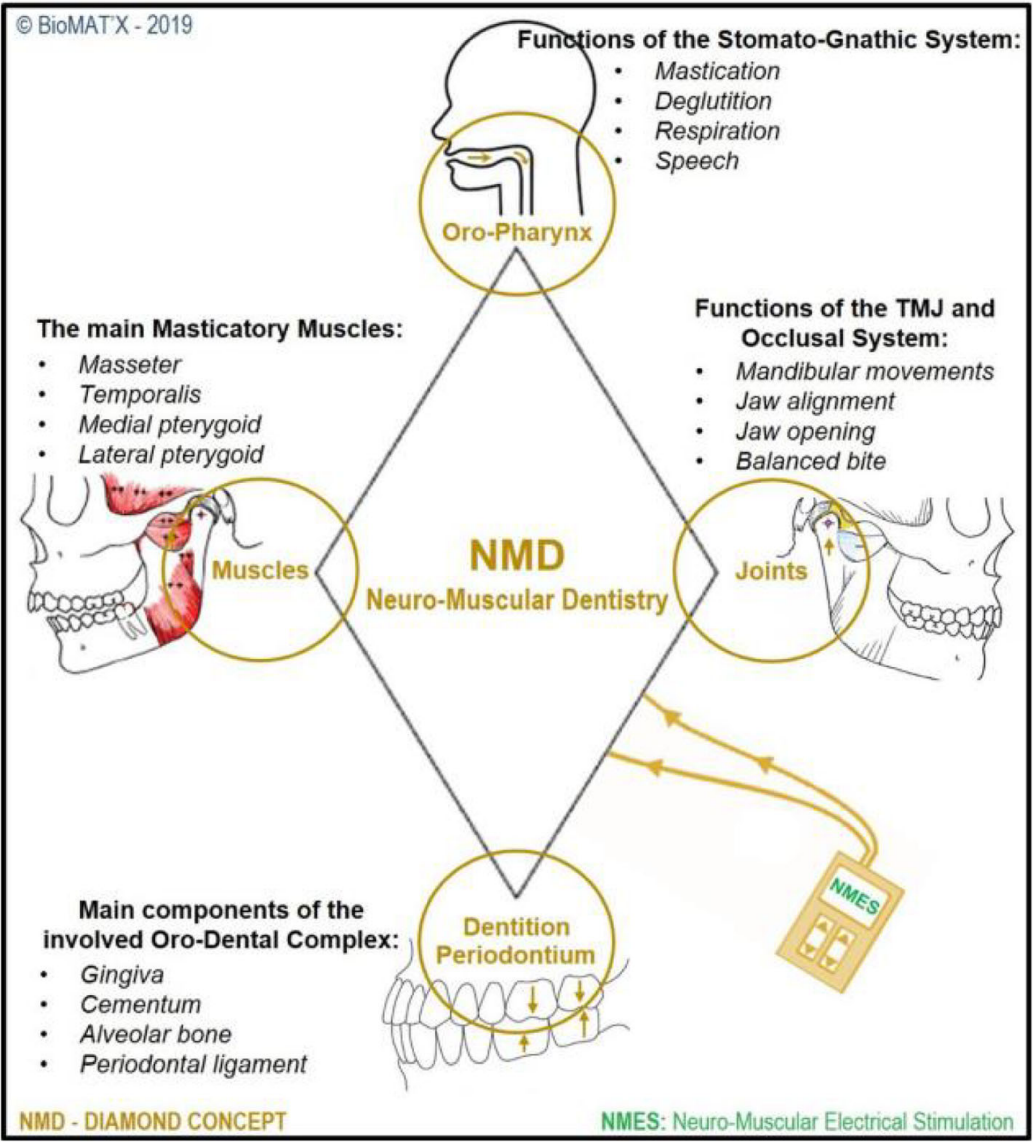
‘Diamond’ concept for Neuro-Muscular Dentistry. The four components of the diamond Oro-pharynx, Joints, Dentition Periodontium and Muscles, and the sub-components of each one which neuromuscular electro-stimulation (NMES) could have positive effects after treatment

Nevertheless, the efficiency and efficacy of the different pharmacological approaches and medication candidates available to date have not been strongly proved and are often associated with poor or subpar clinical results [8]. Endoscopically-injecting Botulinum toxin type A (BoNT-A) into the gastro-esophageal sphincter and upper esophagus to decrease tone and the spasms causing dysphagia, is one example, with short-term/limited improvements in restoring swallowing functions, from our experience. Cystine-depleting therapy with Cysteamine (long-term use in patients suffering from nephropathic cystinosis), is another [8]. This further emphasizes that medical therapy(ies) for deglutition/swallowing difficulties and OPD do have an imperative need for development and innovation. Therefore, the mounting awareness, attention and interest for novel alternative dysphagia rehabilitation modalities is no surprise. Yet, the lack of inter−/intra-disciplinary communication tends to tackle TMDs and OPD separately despite the *possible* causal and sequential relationship between both conditions and their negative impact on oral health-related QoL and over-all QoL. Hence, NMD is suggested to expand and include, besides occlusion and mastication, the complex mechanism of swallowing thereby involving the skeletal muscles of the tongue and smooth muscles of the pharynx and esophagus, a revised ‘diamond’ concept, we propose herein, for NMD from the traditional triad scheme (Fig. 1). It is noteworthy that in this review, radical curative and invasive surgical strategies including vocal fold augmentation (temporary or permanent) and crico-pharyngeal myotomy will not be discussed. We shall focus on sensori-motor stimulation as alternative therapeutic strategies and methods to tackle both, TMDs and OPD. Hence, the purpose is to attempt to raise awareness and initiate bridging the gap between contemporary dentistry, dentists and SLP or Speech and Language Pathologists; an allied health profession providing treatment, support and care for children and adults who have difficulties with communication, eating, drinking and/or swallowing, towards better care for our patients. SLP has been taken care of OPD treatment in clinical establishments and recently there are SLP personnel and teams specialized in managing the symptoms and consequences of TMD through Oro-facial Myo-functional Therapy (OMT).

Undeniably, the effectiveness of neuromuscular electrical stimulation or “NMES” has been under question and development since the late 1980s, mainly for patients suffering dysphagia, until FDA (U.S. Food and Drug Administration) approval/clearance in 2002 upon validating its use for the anterior neck muscles and face in humans as a means of improving swallowing [9–11]. Scientific evidence on safety and efficacy (short- vs long-term) has been mounting since, mainly on normal and disordered swallowing to further elucidate the underlying mechanisms [12]. Furthermore, NMES has been investigated in post-stroke patients as well as in those suffering head and neck cancer, neuromuscular and neurodegenerative/neurological diseases, traumatic brain injury, Sjogren’s syndrome, and respiratory failure, to list a few, among other chronic medical conditions [5, 11, 13]. Today, besides its use in SLP, NMES to improve or restore muscle use is also commonly-practised in physio-therapy and in sports medicine (arthritis, for example) [11]. Briefly, NMES allows an electrical current (flowing from a source - an external device) to alter current intensity or amplitude (through electrodes that are in direct contact with the body) to create a contraction via de-polarizing the nerves (motor end plate) that are responsible for the motor innervation to a particular muscle or to particular muscle fibers, in order to rehabilitate (or re-educate) and augment weak muscle contractions [11]. This, as a result, improves the purposeful movement of structures that are controlled by the stimulated muscles (functional muscle contraction patterns) [11]. NMES can be achieved either trans-cutaneously (surface/superficial) or percutaneously (intramuscular, intrinsic, epimysial) [11]. Surface NMES is of interest, here in, given its established safety and efficacy in strengthening neck musculature and as an *adjunct* modality to treat traditional OPD, especially in post-stroke and traumatic brain injury patients [14].

Recent evidence demonstrated that NMES can also be safely implemented immediately after cardiovascular surgery [15] as well as in patients suffering from severe chronic obstructive pulmonary disease [16] and to improve gross motor function in children with cerebral palsy [17]. Furthermore, for TMDs, NMES can potentially support re-establishing the lost balance between dentition/teeth, joints, muscles and the whole head and neck region, via complementing the traditional regimens including occlusal splints (removable/mechanical, commonly-used for bruxism) and TENS (transcutaneous electrical nerve stimulation or in better words ultra-low frequency electrical muscle stimulation – basically, muscle relaxation via a mild electrical stimulation, normally used to establish the true resting occlusion status); towards a 3-dimensional or even a 4-dimensional contemporary dental practice, in terms of diagnosis and treatment, to better care for our patients and their overall health and QoL.

## Discussion

### Electrical stimulation in the treatment of Oro-Pharyngeal Dysphagia

For many years, electro-therapy has been studied and used in clinical practice by medicine especially in physical therapy to relieve pain using transcutaneous electrical nerve stimulation (TENS), its purpose is to generate analgesia [18]. Another frequent use of the electrical stimulation is to provide muscle strengthening and to promote wound healing by the method called neuromuscular electrical stimulation (NMES). The aim of NMES is to compensate for a temporary or permanent deficiency of voluntary muscular activity and usually is applied in order to increase muscle strength to improve the (active) stability of a joint, recover muscle strength in cases where it cannot be used properly (muscle injuries, fractures) and increase muscle strength to achieve greater and better physical performance, for example, in athletes [18]. In general, all are a low-frequency current designed to produce effects in the targeted area/zone, obtained thru physical, biological and physiological reactions of the treated tissue.

### Oro-Pharyngeal Dysphagia definition

A large amount of research from animal and human models suggest a positive effect of NMES on muscle recovery following injury or disease [12] that is why some clinicians, including speech and language pathologists (SLP), have reached for it in its effort to insert more objective tools in their treatments to complement with clinical interventions. Due to its established benefits, this therapy is often used to treat patients with swallowing difficulties and OPD. As previously-described, OPD is classically defined as a swallowing disorder characterized by a difficulty in the oral preparation of the food bolus and/or in the movement/passing of food (and liquid, including saliva) from the mouth to the stomach, often due to neuro-degenerative, drug-induced or localized structural pathologies such as head and neck tumors, lesions and associated surgical and/or radiation injuries. The alteration ranges from a delay or lack of transfer of the bolus, to an error in the direction of the bolus and its passage to the airway (resulting in tracheal aspiration), with the complications that this entails: in the first case, nutritional alterations, and in the second, respiratory infections [19], such as aspiration pneumonia. Aspiration may lead to necrotizing broncho-pneumonia and lung abscess formation [20]. Ideally a multi-disciplinary team of professionals should be aware of such complications and regularly follow-up the progress of the condition(s). Dysphagia can be a clinical consequence of a broad spectrum of health conditions across the life-span, including pre-maturity, developmental disability, traumatic brain injury, head and neck cancer, neuro-degenerative disorders, and stroke. Also, it is a prevalent difficulty among ageing adults (naturally). The exact prevalence of dysphagia among these populations is not known, although for dysphagia following stroke, it is estimated to range from 13 to 94%. Further, between 27 and 80% of naso-pharyngeal carcinoma patients experience dysphagia following radiotherapy, and more than 80% of patients with the Parkinson’s disease suffer from this swallowing disorder [5]. The presence of dysphagia is also associated with a significant increase in the risk of death, disability, length of hospital stay, bounce-backs and institutionalized care. As afore-mentioned, the overall QoL of these patients tends to decay.

### OPD evaluation and diagnosis

Dysphagia is categorized by the phases of the swallowing that are altered in two: the oropharyngeal dysphagia including the preparatory, oral and pharyngeal phases, and the esophageal dysphagia including the esophageal phase of the swallowing. We shall focus on oro-pharyngeal dysphagia or OPD. The medical history and an accurate clinical examination are critical to identify the volume and viscosity of solids and liquids the patient is having trouble with, which in turn suggests the origin of the dysphagia. Dysphagia with solid indicates obstruction while dysphagia with liquids is a sign of functional dysphagia [13, 18], frequently causing aspiration and repetitive respiratory infections. The most common signs of aspiration are choking, cough and wet voice immediately after ingesting liquids, however, in neurologic patient there is a chance of presenting silent aspiration. The sensation of residue in the pharynx leads to pharyngeal hypo-motility, frequent in neuro-degenerative diseases. Other signs of dysphagia are increase in the time in every meal and a recent loss of weight.

The parameters to diagnose dysphagia are the effectiveness of swallowing or the ability of the patient to ingest all of the calories and water needed to be well nourished and hydrated, and the safety of swallowing or ability to ingest water with no risk to produce respiratory problems. To determine the mentioned above, different scales and screening tools exist to assess risk (early) and rate severity according to the degree of disruption of the structures involved in swallowing and assist in the recommendations of the type of oral intake necessary to ensure/promote safe swallowing, both measured by instrumental evaluation, “Dysphagia Rating Scale” (DRS) and “Functional Oral Intake Scale” (FOIS), respectively. Another assessment tool is the using video-fluoroscopy, which allows the evaluator to watch the complete process of deglutition, from the oral to the esophagus phase, and to determine the temporal measurements (oral transit time, pharyngeal delay time and pharyngeal transit time) of the swallowing. Rehabilitation Swallowing Therapy (RST) conducted by SLP has to correctly evaluate and identify the signs and symptoms that properly or accurately characterize the condition of the patient to proceed with treatment and to guide the medical treatment if/when necessary. For example, Azzolino et al. [21] recently studied and reviewed “*sacropenic dysphagia*” or the association between sacropenia (the progressive and generalized loss of skeletal muscle mass), ageing, physical status (disability versus exercise), and swallowing function. The authors highlighted the importance of using dysphagia screening and assessment tools, such as the EAT010, a 10-item Eating Assessment Tool, (often employed alongside a water test and combined with pulse oximetry), especially when performed early, on the management and treatment of both conditions: sacropenia and dysphagia, and the resulting systemic benefits from promoting a healthy life-style in this patient population [21].

### OPD treatment(s)

OPD may be life-threatening, particularly for the elderly/geriatric and the frail/institutionalized patients. Management (interventions and treatment) can be as well. Due to the high-risk complications of OPD and often co-presence of other disorders, diseases and conditions, it requires the care of a multi-disciplinary team that should include various professional profiles: nurses, speech therapists or SLPs, dieticians, gastro-enterologists, oto-rhino-laryngologists, radiologists, rehabilitators, geriatricians, neurologists, digestive surgeons when necessary and the participation of an oral and maxilla-facial surgeon when the oro-dental and maxilla-facial structures are involved and/or compromised. To manage/treat oropharyngeal dysphagia, there are several therapies that, taken together, have the common goal of reducing the morbidity and mortality associated with respiratory infections, improving the nutritional status and trying to get the patient to return or maintain a normal oral diet (oral-intake) [19]. The best way, thus far, to approach therapy is to make decisions based on the severity of the swallowing alterations, identified during the clinical screening and evaluation, and the safety and effectiveness of the available procedures/regimens. One of the first steps of the therapy is to change the volume/size and viscosity of the diet/food, to generate a proper alimentary bolus and subsequent bolus flow. Slowing the rate of chewing/mastication, eating and swallowing of the smaller-sized bolus follows. Then the need for postural changes, rehabilitation maneuvers and augmentation of oral sensitivity are evaluated and considered for the patients in a more severe state. Indeed, maintaining a proper head posture while eating, drinking and swallowing is important, and there are special tools and utensils such as modified cups (without rims), straws, narrow spoons and shallow bowls, to aid and prevent a back-ward head tilt that would result in extending the neck and can therefore misdirect the entry of food or liquid into the airway and consequently lead to choking. SLP and occupational therapists are often involved herein in suggesting the suitable swallowing aids and providing adaptive training for the proper use of the alternative utensils.

In cases where oral intake is not possible and nutrition is considered inadequate, patients (such as in the case of a cerebral vascular accident or traumatic head injury) will require an alternative, safe, yet long-term and more invasive way or method of feeding and hydration, referred to as enteral feeding or nutrition. Such include, besides nutritional supplements, the use of a naso-gastric and naso-jejenal tubes (can require surgical placement) or percutaneous endoscopic gastrostomy or PEG, depending on the severity of the disorder and symptoms. The clinical decision also will weigh in the advantages and possible complications for each route of enteral nutrition [13, 18, 19]. Such a topic continues, to an extent, to be debatable, especially in head and cancer patients, when considering mortality rates, weight gain/loss, muscle atrophy, length of intervention and psycho-social impact (including activities and hobbies with family and friends), risk/incidence of tube failure due to blockage or leakage and subsequent interruption in feeding and hydration, to mention a few. Nutrition, in general and/or if eneteral in specific and whether prescribed for short- or long-term, is to be monitored by dieticians to determine and monitor the caloric and protein requirements for every patient. Alongside, rehabilitative regimens, including oral motor control, swallowing maneuvers (effortful swallow, super-supraglottic swallow, Mendelsohn maneuver, among others), swallowing exercises and thermo-tactile stimulation(s) can be implemented to increase or enhance the strength and mobility of the structures involved in swallowing (lips, masticatory muscles, tongue, soft palate, larynx and glottic closure). The combination of strategies and modalities will depend on the status and necessities of each patient. Hitherto, efficacy of such protocols is moderate and outcomes are often doomed by vague prognosis. It is noteworthy herein that any and all interventional decisions must respect patient autonomy.

### Effect of NMES in OPD treatment

With the intention to complement the RST a novel NMES treatment for OPD was officially included in 2001, when the U.S. Food and Drug Administration (FDA) approved the use of a non-invasive electrical stimulation device called VitalStim (Chattanooga Group, Hixson, TN, USA). However, currently the controversy of this therapy is still on the table due to different factors including the persistent lack of information about how the NMES acts on and stimulates the muscles responsible for swallowing, and to the existent discrepancies in the reported effects of NMES, even though the literature and use of NMES in limb rehabilitation is extensive. Yet, VitalStim remains the only FDA-cleared device for treatment of dysphagia.

Many authors consider the NMES therapy effective to treat OPD in stroke patients. A preliminary meta-analysis revealed a small but significant summary effect size for transcutaneous NMES for swallowing disorders (*p* < 0.05) including stroke, cancer, head trauma, and respiratory failure [12]. Permsirivanich et al. [10] compared treatment outcomes between RST and NMES intervention in stroke patients with oropharyngeal dysphagia. Overall results show an improvement in the 91,30% of the patient’s FOIS by at least in one unit compared to the initial scores. Specifically, the average changes in FOIS scores were 2.46 + 1.04 for the RST group and 3.17 + 1.27 for the NMES group, which was statistically significant (*p* < 0.001) [10], showing a greater improvement obtained with the NMES therapy. Supporting this idea, Terré and Mearin [19] conducted the first prospective randomized study, double-blinded, with subjective and objective evaluation and standardized (1 and 3 months of follow-up), in patients that suffered brain injury, but with a different approach, they compared two groups, one receiving NMES and RST, together, and the other group received sham electrical stimulation and RST. The results demonstrate that NMES, combined with conventional swallowing therapy, significantly accelerates swallowing function recovery in patients with acquired brain injury (stroke and severe traumatic brain injury), since, an improvement in FOIS scores and temporal measurements, specially the oral transit time, were observed. The authors also affirm that the technique is well tolerated and free of adverse effects [19]. A pilot study evaluating the effect of NMES on swallowing in 10 elderly with stroke sequelae shows that immediately after the therapy and after 3-months period there is an improvement in the parameters assessed such as Dysphagia Outcome and Severity Scale (DOSS), Penetration Aspiration Scale (PAS) and Quality-of-life protocol, although no changes were significant in FOIS results [22]. The authors concluded that in short- and mid-terms, NMES therapy results in a decrease of dysphagia and improves QoL. Also, few studies determine the specific effects on swallowing physiology suggesting that the NMES improves laryngeal elevation and swallowing delay [10, 23] and enhances sensory feedback [23]. Recently, improvement in the aspiration status was seen in 83.3% of medically-complex children who underwent NMES as a component of their feeding therapy [24].

On the other hand, Tan et al. [5] conducted a meta-analysis to assess the overall efficacy of NMES and RST. The results showed that NMES is more effective for the treatment of adult dysphagia patients of variable etiologies (such as head and neck cancer, radiation damage and Parkinson’s disease) than RST, but not in patients with dysphagia after stroke, the effectiveness of NMES and RST was only comparable [5]. Some authors relegate it, based on the belief that it increases muscle volume but does not return to the functional muscle and interferes with the normal regeneration of the nerve, which favors aberrant reinnervations [25], in the case of facial paralysis patients. In a large randomized controlled trial investigating the effect of NMES in the treatment of post-irradiated patients with head and neck cancer with dysphagia the results did not show improvement in the swallowing performance of the patients after 12-week treatment program [26]. Overall, the discrepancy between the results of the studies could be explained by possible methodological flaws such as lack of randomization and use of non-blinded observers to the outcomes, failure to include a control group, among other bias in some of the studies (see Table 1), and also these studies could reflect the real limitations of the electrical stimulation uses. Also, the spontaneous recovery, resolved in the majority within 2 weeks after stroke, may exaggerate the function of NMES is hardly considered.

**Table 1:** Summary of Selected Studies Employing NMES in the Treatment of OPD

Ref	Study Design	Participants (SS; Age; NMES / RST; etiology)	Intervention Type	Outcome Measure	Therapy Duration
[10]	RCT	23; 64.5 ± 8.8 / 64.73 ± 9.39; 9/14; Stroke.	NMES vs. RST	FOIS	1 h/day, 18 sessions. Around 20 days.
[13]	RCT	60; NMES 31 / Control29; 56.50 ± 8.73; 55.83 ± 7.97 15/16, 14/15; Nasopharyngeal Carcinoma.	NMES, balloon dilatation vs. Control	WST VFSS	1 h, 5 days per week, 4 months.
[19]	RCT	40; mean age of 48 years; 12 / 8; Stroke.	NMES, RST vs. SES, RST	FOIS	1 h (45 min of NMES), 20 sessions. At least 1 month (3 months follow-up).
[22]	CCT	10; Elderly; Stroke.	NMES, OST vs. OST, sham-NMES	FOIS PAS DOSS QoL protocol	2 stages of 10 min NMES with exercises. Thrice a week for 4 weeks (3 months follow-up).
[25]	CCT	99; 75, 5/78, 1; 53 / 46; Stroke.	NMES vs. RST (thermal-tactile stimulation)	Swallow Score System	1 h/day, variable sessions. Did not limit duration (follow-up 3 years).
[26]	RCT *double-blinded*	170; over 21 years old; Head and Neck Cancer.	NMES	PAS VFSS	3 training sessions and at home 2 times a day, 6 days a week, for 12 weeks.
[27]	RCT	20; Children; Cerebral Palsy.	NMES, OST vs. OST, sham-NMES	ASHA – NOMS	20 min of NMES after OST. Twice a week for 8 weeks.

Legend: *SS* sample size, *RCT* randomized controlled trial, *CCT* clinical controlled trial, *RST* traditional rehabilitation swallowing therapy, *WST* water swallow test, *SES* sham electrical stimulation, *OST* oral sensorimotor treatment, *FOIS* Functional Oral Intake Scale, *DOSS* Dysphagia Outcome and Severity Scale, *VFSS* video-fluoroscopic swallowing study, *PAS* Penetration Aspiration Scale, *ASHA – NOMS* The American Speech Language Hearing Association (ASHA)‘s National Outcomes Measurement System (NOMS)

### NMES controversy

**Fig. 2:**
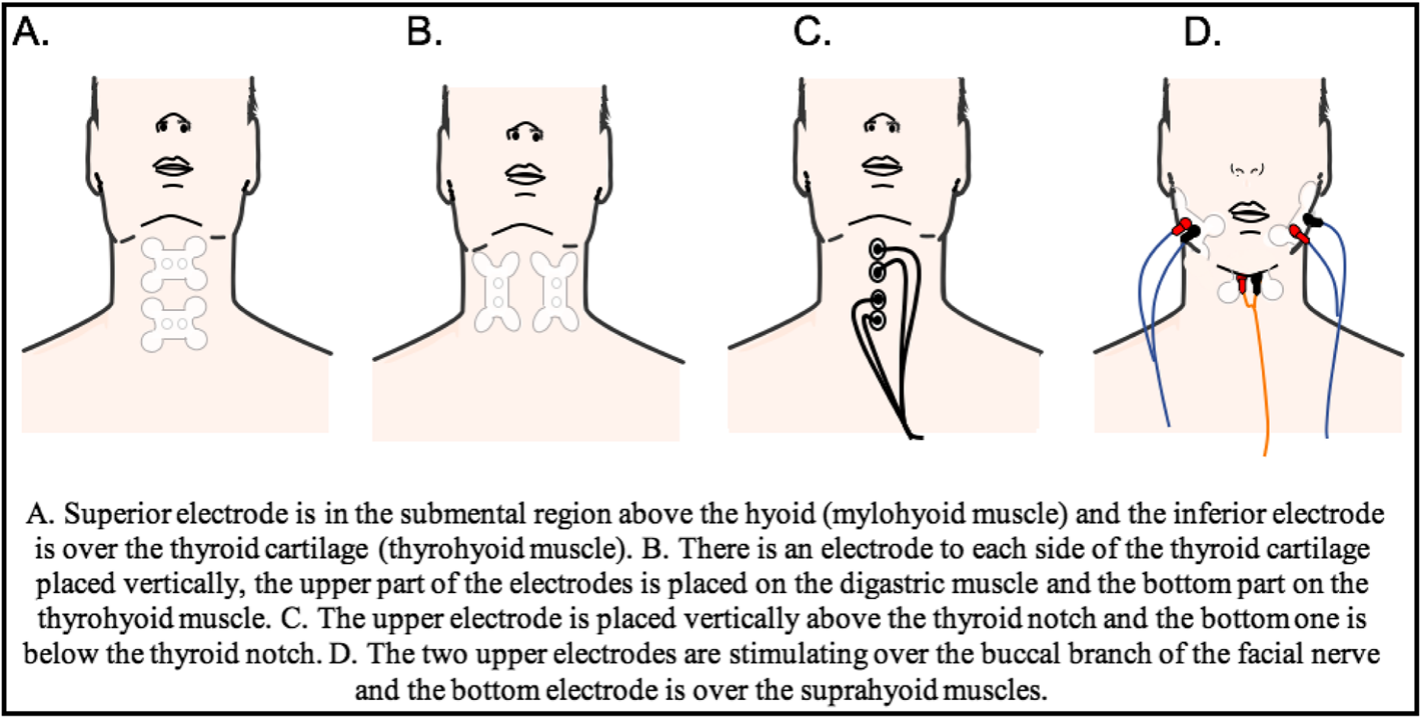
Examples of the placement of electrodes during a Neuro-Muscular Electro-Stimulation (NMES) treatment for Oro-Pharyngeal Dysphagia (OPD)

Despite that there is no convention on how or when to use the NMES treatment, and considering that there are some of the studies selected for this discussion that didn’t specify the placement of the electrodes, is relevant to note that the manufacturer of the electrical stimulation device approved by the FDA suggests applying the treatment for dysphagia at least twice a week at the beginning, localizing carefully the electrodes in different positions on the anterior neck area (see Fig. 2). During sessions of electro-stimulation, the patient swallows when stimulation is on after the parameters of the current are modified to (1) make the stimulation comfortable for the patient (intensity) and to (2) set a protocol of stimulation (frequency, work time, rest time) according to the patient’s needs based on the underlying pathology. To other author the treatment sessions continues until the patient’s improvement plateaus [25].

The latent controversy about this subject that prevents clinicians to use NMES might be caused by a lack of adequate high-quality research or the uncertainty of potential disadvantages. Those are some of the reasons discussed previously and that were also given into a survey of a small sample of SLPs in Iowa showing that nearly 3/4 of these respondents indicated that they were unsure if NMES was effective, and > 60% indicated that they were unsure if the literature supports its use. The results of the survey highlight that although many clinicians are aware of potential functional benefits of NMES, misconceptions persist related to benefits in swallowing physiology and treatment complications [28]. Add to that, in the literature about this technology almost every study NMES is complemented with traditional OPD treatment by a SLP, most of them differ on how the therapy is conducted having different current’s amplitude and dosage of the treatment in terms of how many weeks, how many days per week and for how long the current is applied going from 20 min up to an hour every session, and also some authors mentioned that the severity of the OPD might impact in the NMES therapy results, improving function when the dysphagia is mild to moderate and having almost no benefits when dysphagia is severe [10, 23], variables that could bias the results of the electrical treatment itself when not considered into the study.

As said before OPD is present in many health conditions where also NMES has been studied with various results, such as Multiple Sclerosis, stroke, traumatic injury, Parkinson’s disease, muscular atrophy, and a number of other neurologic diseases. Long & Wu [13] concluded that the combination of electrical stimulation and conventional rehabilitation treatment is superior to conventional RST alone in patients suffering from OPD following treatment for head and neck cancer [13]. Other studies demonstrated that oral sensorimotor treatment and NMES facilitated swallowing functions in children with cerebral palsy and dysphagia [27] and in stroke patients, electrical stimulation helped restoring 45% of all swallowing severities in a week with one-hour daily treatment [29]. These variety of studies indicate that the NMES could be versatile in the clinical practice and even in other areas extrapolating its use to obstructive sleep apnea or amyotrophic lateral sclerosis (ALS), therefore this treatment could be fair complement to the traditional therapy rather that an alternative therapy, as suggested many studies.

Some others interesting applications of this technology related to orofacial conditions are the treatment of obstructive sleep apnea (OSA) and xerostomia. The prevalence of OSA has increased over the years associated to obesity and as an isolated condition manifesting deterioration in the quality of life and even causing brain damage specifically by a functional alteration of blood-brain barrier (BBB), as shown in a recent study that shows significant changes of BBB integrity in OSA subjects [30]. The standard treatment is the use of continuous positive airway pressure therapy (CPAP) which ensures a permeable upper airway, but the adherence to this treatment is limited due to some discomforts while wearing it [31]. Therefore, other invasive and non-invasive treatments are available. In the first group there is a newly treatment modality consisting of implanting an electrical-stimulator to stimulate contraction of upper airway muscles during the night called selective upper airway stimulation (sUAS) or hypoglossal nerve stimulation. A longitudinal study shows that this treatment improves sleep apnea conditions, reduces daytime sleepiness, has a high adherence to the therapy and the patients expressed a positive attitude towards sUAS [30] in agreement to other studies that showed along with therapeutic benefits (e. g. improvement on Apnea-Hypopnoea Index (AHI)) an acceptable risk and tolerance [32]. Several devices have been launched with different mechanism to prevent muscle fatigue, to synchronize with respiration and even different methods of stimulation selecting portions of hypoglossal nerve and/or genioglossus muscle [32]. These devices have been tested by feasibility studies demonstrating this technique to be safe and efficacious, and currently there is one of them approved by FDA.

On the other hand, neuro-stimulation has also been used as a non-invasive treatment for OSA such as percutaneous electrical stimulation of the genioglossus and transcutaneous submental and intraoral electrodes. However, the effectiveness of this approach by itself is not certain, although they show a change on pharynx structure and muscles no significant improvement on AHI is accomplished [31]. These results could respond to the several limitations on the studies and to the characterization of the patients, because of the different zones of collapse in the pharynx, the amount of adipose tissue, anatomic abnormalities among other factors. Some authors suggest that the combination of this therapy and CPAP could reach better results [31]. The same observation has been done for the myofunctional therapy of mild–moderate OSA which has the objective to promote tone changes in the upper airway musculature so the risks of collapsing during the night is reduced. In a review on this matter is described how myofunctional therapy improves AHI, daytime sleepiness, and quality of life also reduces volume of specific structures and fat in the upper airway muscles [33]. More studies are needed to verify the results over time and the direct relationship myofunctional exercises and significant changes in the pharynx structures [33]. Also, NMES has been recently studied as an alternative therapy for patients with intermittent claudication (IC), common condition in peripheral artery disease (PAD). The first part of the research, a proof-of-concept study, was conducted displaying a 6-week treatment while the patient had to use a footplate NMES device daily for 30 min. The results showed that NMES can improve functional capacity for both primary outcomes maximum claudication distance (MCD) and the initial claudication distance (ICD) in patients with IC. The RCT study later used the same treatment protocol and found a significant adjunctive benefit of NMES to supervised exercise program alone for ICD. The QoL survey results also improved significantly in both studies when using/applying NMES [34].

Now that more research has been done to explain the effects of NMES on the muscles other benefits have been found as this method increases the responsiveness of corticospinal pathways and that this effect is different depending on the muscles, enhances sensory feedback and promotes more global adaptations than those expected from the contractions evoked in the target muscle allowing this treatment to augment activation signal generated by the nervous system in healthy individuals and clinical cohorts [35].

Currently, electrical-stimulation is also studied to treat xerostomia mostly through transcutaneous electrical nerve stimulation (TENS), that promotes analgesia mainly. The objective of this therapy is to increase salivary flow. Dalbem et al. [36] applied TENS in the region of parotid and submandibular salivary glands for 20 min to patients that had radiotherapy-induced xerostomia resulting in a significant increase of salivary flow. Authors suggest more studies on the mechanism of action of TENS in the salivary glands are needed.

It should be noted that no studies reported complications from the application of NMES in adults with OPD or OSA, however for pediatric population poor and limited evidence supports the treatment [37]. The development of electrical stimulation devices these days allows the clinician to modify the setting to the needs and comfort of every patient individually, even including biofeedback systems to facilitate the performance of the patient, these improvements are due to the experienced advocates that have seen improvement in their patients using NMES [10, 12, 19]. These are some of the reason to conduct more high-quality studies about the efficiency of this rehabilitation tool.

### Electrical stimulation in Temporo-mandibular disorder therapy (TMD)

Swallowing is only one of the 5 different functions that the stomatognathic system fulfills, including breathing, suction, chewing, speech and swallowing which could be altered by many conditions impairing the correct functioning of the orofacial structures. One of the most common complaints presented to dentists is facial pain that sometimes comes along with muscle spasms and presence of joint noise (clicks), some of the signs and symptoms described for Temporo-Mandibular Disorder (TMD).

### TMD definition, signs and symptoms

According to the American Academy of Orofacial Pain, the term temporomandibular disorder refers to a set of clinical problems that involve the masticatory musculature, the temporomandibular joint (TMJ) and associated structures, or both, being identified as the leading cause of non-dental pain in the orofacial region and is considered a subclass of musculoskeletal disorders [38]. Affecting approximately 5 to 12% of the population, TMD is the second most common musculoskeletal condition (after chronic low back pain) resulting in pain and disability [39]. Known for been more prevalent in women, considering that 8–15% of women are diagnosed with TMD and men only 3–10% [40]. TMD is a multifactorial etiological disorder with various factors such as psychological factors, macrotrauma, and microtrauma responsible for it [2], affecting the structures and function of any of the elements that support and compound the complex mandibular movement, including TMJs, masticatory muscles and the occlusal surfaces of teeth. This disorder has also been associated to comorbid factors such as bruxism and occlusal instability [2, 41], which are still not certain whether they are directly related to the development of TMD. The signs and symptoms most commonly described by the literature are joint pain, pain on palpation, headaches, movement-induced fatigue, limited mandibular movement, disc displacement, alteration on neuromuscular activity of masticatory muscles and otologic symptoms like tinnitus affecting the individual’s quality of life.

### TMD evaluation and diagnosis

Diagnostic criteria for TMD with simple, clear, reliable, and valid operational definitions for the history, examination, and imaging procedures are needed to render physical diagnoses in both clinical and research settings [39]. This is no easy task, considering that (1) one of the classic symptoms, orofacial pain, could also be caused by many other pain conditions, (2) that up to 93% of the population report at least one of the signs and symptom [42], and that (3) there is a controversy on the direct relation between this disorder and orthodontics [43], very common procedure these past years. These is why the professionals provide themselves with a fair amount of diagnostic tools such as electromyography (EMG) to study the status of the muscle neuromuscular activity; Cone-beam Computed Tomography (CBTC) to evaluate upper airway, cervical vertebra, TMJ morphology, and condylar position; Electrosonography (ESG) to analyze frequency and amplitude of the noise and the position of the condyle during the opening/closing of the jaw at which sound is produced; Computerized Mandibular Scanning which is a jaw tracking device identifying the relationship between the mandible and skull and the delicate functioning movements of the jaw with accuracy up to one tenth of a millimeter [1, 2], ensemble with protocols to correctly identify TMD. Recently, the most widely employed diagnostic protocol for TMD research since its publication in 1992, the Diagnostic Criteria for Temporomandibular Disorders (DC/TMD) [44] which features two parts: Axis I (clinical TMD conditions) and Axis II (pain-related disability and psychological status), has been expanded in 2015 in Axis II protocol by adding new instruments to evaluate pain behavior, psychological status, and psychosocial functioning [39]. A correct diagnosis is fundamental to determine the following treatment, therefore, to identify the localization of the pain, the presence or not of disc displacement and others symptoms is relevant when choosing the different treatment alternatives and combinations.

### TMD treatment(s)

In 1960, Travell [45] described that involuntary shortening of one or more of the masticatory muscles may cause an eccentric position of the mandibular condyle, disorientation of jaw movements, and restricted opening of the mouth [45], summarizing the current challenges for TMD treatment besides the orofacial pain. The goal of treatment for TMDs is elimination or reduction of pain and a return to normal TMJ function [46]. In general, the treatments could be categorized into two groups, (1) supportive therapy and (2) definitive treatments [46]. Definitive treatment aims at eliminating the etiological factors, and on the other hand, supportive therapy directs toward alleviating the symptoms [2]. As a convention between clinicians’ practice and suggested in every article revised the supportive therapy is done first and if the clinical symptoms don’t improve then the definitive treatments come into consideration, since surgery is not reversible. Within the first treatment considered are pharmacotherapy, physical therapy, hyaluronic acid (HA) joint injection and botulinum toxin (BTX) type A injection [47]. A frequently used device in the TMD’s management are interocclusal appliances or occlusal splint to relieve symptoms and to achieve a proper jaw relationship [48], usually used during the night. These have to be complemented from the first moments of the therapy with patient education about their diagnosis, their role in the treatment meaning self-management approach, avoiding harmful oral behaviors, and to control stress and to promote relaxation [49, 50]. Another very important factor to consider is the cervical and body posture that may help to reduce symptoms [49], due to the relation between the muscles from different parts of the body specifically to the TMJ through muscle chains that compensate when one component is unbalanced. On the other hand, there are the not reversible treatments, where we found that the most common surgical procedures are arthrocentesis and arthroscopic treatment [46], only used for internal derangements of the TMJ. Also, permanent mandibular repositioning has been used as preventive and managing approach, which is questioned as a medical necessity, arguing that it has irreversible consequences in the articular condyle and that today’s supportive therapy could provide successful results with low risks [48].

### Effects of electrical stimulation in TMD therapy

Considering that complex normal movements of the joint require an efficient and relaxed musculature physical therapy plays an important role in the TMD treatment, which considers low-level-laser therapy, ultrasound, manual mobilization, massage, jaw exercises (e.g. relaxation, rotation, stretching, isometrics and postural), application of superficial heat and/or cold and electro stimulation. The electrical stimulation devices have two main purposes for the treatment of TMDs: relief of pain and relief of muscle hyperactivity or spasm [46]. The most common electrical stimulation used for this purpose is transcutaneous electrical nerve stimulation (TENS). This is a safe battery-operated device delivers a mild electrical stimulus to the muscles via neural pathways. The stimulus induces involuntary contraction of the muscles controlled by the facial (7th) and masticatory (5th) cranial nerves promoting anesthesia.

In dentistry, TENS has been used for pediatric treatment in procedures such as minor extractions, restorations, and pulp therapy; in adult patients it has been used successfully as an excellent analgesia during various procedures like rubber dam placement, cavity preparation, pulp capping and other endodontic procedures, prosthetic tooth preparations, oral prophylaxis as well as extractions; and also TENS is used in chronic pain of maxillofacial pain like trigeminal neuralgia, post herpetic neuralgia and TMD [51, 52]. In this article we shall focus on the TMD applications of TENS. Rodrigues, et al. evaluated the effect of TENS on pain and electromyographic (EMG) activity of the jaw elevator muscles in TMD patients compared to a control group with no signs or symptoms of TDM. They found that TMD subjects present higher electromyographic activity with the jaw at rest than clinically normal subjects, as one of the symptoms of the syndrome and after the application of the treatment they concluded that a single application of TENS is sufficient to relief of pain. However, in general, there was no significant reduction of electromyographic activity of the studied muscles [52], so the effects of the stimulation on the muscle was not evidenced through EMG, even though, the patients manifested relief of pain.

Due to the uncertainty about the cause-effect relation between bruxism and TMD many authors have tried to answer the question. In 2002 Alvarez-Arenal et al. [53] evaluated the action of an occlusal splint with TENS upon the manifestations of TMD in patients with bruxism through a crossed-design experimental study with a total of 24 individuals with an average age of 36,5 years (15 males and nine females). They could determine that over 60% of the bruxing patients presented TMD indicating an association between bruxism and TMD. They also found that in this study the occlusal splint treatment and TENS failed to improve the signs and symptoms of TMD in the bruxing individuals [53]. Occlusal splints are the most traditional treatment for bruxism and TMD, although its efficiency has been questioned lately claiming that this device may lead to the development of TMD in some patients [54], however, these signs are most likely transient. Overall, clinicians treating TMD accept the use of occlusal splints to prevent tooth damage and also promote the use of TENS based on the evidence and the experience that this electrical stimulation helps relief TMD symptoms.

### TMD myo-functional performance and SLP treatment

Contributing to the supportive therapy and with the purpose to the relieve of pain and to facilitate the execution of stomatognathic functions of TMD patients the speech and language pathologists (SLPs) work with the neuromuscular system to reestablish the harmonious functional balance of chewing, swallowing and speech. To plan the treatment the components of the stomatognathic system, the overall posture, the occlusion and the functions have to be evaluated in first place. The protocol of orofacial myofunctional evaluation with score (OMES) is widely used to determine lips, tongue, jaw, and cheeks appearance/posture, mobility and performance during deglutition (liquid and solid) and mastication functions [55]. The muscular activity of the masticatory muscles is evaluated by electromyography (EMG), especially for research purposes. In general, individuals with TMD often limit mouth movements and/or perform them slowly when compare to healthy people [56]. It has been observed that these patients showed different masticatory patterns, not exactly meaning presence of alterations in the muscular activity. According to Berretin-Felix et al. [57], who found that the duration of the period of muscular contraction and duration of masticatory cycles are increased in the group with TMD compared to a control groups without TMD. However, alterations were not found in the muscular activity of individuals with TMD [57]. Ten years later, Rodrigues et al. came to a similar conclusion. They found that TDM patients show higher masticatory efficiency, more chewing strokes, increased chewing time and increased EMG activity of masseter and temporalis muscles. Subjects also featured an altered chewing pattern. The authors concluded that patients with TMD showed a changed chewing pattern (uni-lateral mastication, fatigue), yet without impairment of masticatory function [58], meaning that the masticatory performance was efficient after all. A different position is taken by Gilheaney et al. [59] establishing a relation between OPD and TMD through a survey answered by 178 individuals with TMD. It was found that signs and symptoms of OPD are frequently reported by adults with TMDs such as dysphagia for liquids (28%); difficulties chewing hard food (89%); difficulties chewing soft food (58%). Additionally, weight loss attributed to OPD was experienced by 47 respondents (26%) and almost one half of the participants reported anxiety, embarrassment, and subsequent social withdrawal because of TMD-related OPD. The discrepancy in these studies shows that there is a lack of research regarding the masticatory patterns as a factor that causes, maintains or aggravates the condition. At the same time, this exhibits that the research in this field is fairly new, explaining or signifying why we found more descriptive studies than others (see Table 2). This last observation also applies to speech. It has been detected that speech movement patterns are also changed, Bianchini et al. [60] found that TMD patients seem to show (a) a reduction in the maximum vertical opening during speech (relative to that described by dentists with years of experience with the patients); (b) a reduction in the maximum retrusion amplitude, when compared to asymptomatic individuals [58]; and (c) patients present an uni-lateral deviation of mandibular movements during speech instead of a bi-lateral deviation (demonstrated in asymptomatic individuals). All three alterations might indeed impact and damage speech articulation and voice quality.

**Table 2:** Summary of Selected Studies Employing NMES in the Treatment of TMDs

Ref	Study Design	Participants (SS; Age; Gender)	Intervention Type	Outcome Measure	Therapy Duration
[52]	Descriptive	35; TMD patients (23.04 ± 3.5) and NSS patients (23.3 ± 3.0); all Female.	TENS TMD (19 subjects) vs. Control (16 subjects).	EMG VAS	Applied once to each group for 45 min.
[53]	Descriptive	24; average age of 36.5 yrs.; 15/9.	Occlusal splint vs. TENS A resting period of a month and a half between the two treatments– the order of the treatments being randomized for each patient.	PRI Self-reported pain and / or tenderness score	Patients wore the splint 24 h a day, except during meals, for a period of 45 days. TENS session lasted 45–60 min, 15 sessions (one every 2 days).
[56]	Descriptive	40; TMD: aged 17–60 yrs. (mean age of 28 yrs) and NSS aged 22–31 yrs. (mean age of 25 yrs); TMD 4/21 and NSS 2/13.	Masticatory function description. TMD (25 subjects) vs. Control (15 subjects).	EMG	–
[57]	Descriptive	52; aged 18–60 yrs.; paired by age and gender.	Masticatory function description. TMD (27 subjects) vs. Control (25 subjects).	EMG CE of habitual chewing Analysis of ME	–
[59]	Descriptive	135; 18–57 yrs.; both groups in a proportional relation with regard to age and gender.	Speech pattern description. TMD (90 subjects) vs. Control (45 subjects).	EMG	–

Legend: *TMD* Temporo-Mandibular Disorder, *NSS* no signs or symptoms patients, *EMG* electromyography, *VAS* visual analogue scale for pain evaluation, *PRI* pantographic reproducibility index; (−) = does not apply; *CE* clinical evaluation, *ME* Masticatory efficiency

After evaluating the patient, throughout anamnesis and examining the patient (application of protocol of orofacial myofunctional evaluation) to identify the musculature, TMJ and skeletal integrity, and to determine the severity of the condition along with been aware of the odontological behavior, the SLP will proceed with the treatment informing and educating the patient about his/her condition and teaching functional and protective modification. The Orofacial Myofunctional Therapy (OMT) consists of exercises directed to tongue, jaw, soft palate and lateral pharyngeal wall, including adequate functioning of suction, swallowing, chewing, breathing and speech. In general, the treatment consists of thermotherapy, cryotherapy, massage therapy, muscle elongation, myotherapy and specific functional therapy [59]. According to the diagnosis and to objective of the therapy the recommended exercises are isotonic, isometric, isokinetic or with mandibular movement control. Equally important is the functional training of the neuromuscular stability achieved by the exercises named above. In TMD, specifically, the balanced jaw movement during function is to be achieved, therefore, the masticatory musculature has to reach functional balance between the jaw elevators and jaw depressors to avoid jaw deviations and pain. Due to the automatic characteristic of stomatognathic functions a proprioceptive approach is necessary to modify the altered functions [61]. The therapy progresses from achieving that patient’s awareness of the muscular performance to the automating of the correct functional patterns.

Even though the dentists are not well aware of the objectives and benefits of this therapy [62, 63] evidence show that OMT had positive effects in treated patients such as a significant reduction of pain sensitivity to palpation of all muscles studied (but not for the TMJs), increased measures of mandibular range of motion, reduced frequency and severity of signs and symptoms, increased scores for orofacial myofunctional conditions according to OMES [63], and reduction of otologic symptoms [64]. The scientific evidence, the clinical experience and the complexity of the TMD are showing the necessity of a multidisciplinary approach of the individuals with TMD, where different specialties could converge and collaborate including NMD and OMT, along with new technologies, like electrical stimulation such as TENS, as mentioned before, or even NMES. To the best of our knowledge, NMES has not been tested in TMD rehabilitation, but by understanding how this system works and how do masticatory muscles coordinate to procure a correct functioning, both explained in this article, one can see that NMES could help meeting a neuromuscular balance under the logic of reciprocal innervation, knowing that the stomatognathic functions are possible due to the coordinating jaw elevators and depressors and associated muscles in synergistic and antagonistic actions [56], meaning that NMES could active jaw depressor muscles to compensate the jaw elevators muscles (masseter and temporally muscles) action that are usually over contracted in TMD patients [57].

Furthermore … this is a new line of research that could take place in a near future, as others research that are emerging around the different electrical stimulation uses. In 2017, Snyder et al. [64] used electrical stimulation to mimic the transepithelial potentials that occur during the granulation phase of wound healing, and found that HDFs (Human Dermal Fibroblasts) increased random migration behavior within in vitro model under some electrical stimulation conditions even after 10 min, providing a mechanism to enhance wound healing [60]. Direct ES has also been used to enhance the number and size of acetylcholine receptors (AChR) clustering increasing its availability for neuromuscular junction formation using in vitro model [65]. The beneficial effects of ES in promoting osteogenesis were described by in vitro model in a comprehensive study [66]. The results show that the gene expression of some transcriptional factors and proteins that are fundamental to promote osteogenesis and bone mineralization are higher in quantity and had longer time of expression in ES groups than the controls, demonstrating that ES treatment directly up-regulates osteo-differentiation genes. This capacity of ES was also found in a study that tested a conductive graphene-cellulose scaffold integrated with an ES device to treat human adipose stem cells, where these cells had significantly increased proliferation and osteogenic differentiation when stimulated electrically for 21 days compared to control samples with no ES treatment [67].

A in vivo model demonstrated that a chronic NMES treatment exhibits a positive role in protecting muscle from atrophy by significant increasing of miR-1 and miR-133 expression that reduced significantly the expression of pathways that lead to a change in the fiber muscle from fast to slow therefore to atrophy, a skeletal muscle dysfunction common in Chronic obstructive pulmonary disease (COPD) patients [68]. Another set of studies, are testing ES in vivo in rabbits [69] and dogs [70, 71] by fracturing the tibia of these animals but with different conditions of treatment, despite that, both could conclude that the outcomes where positive to increase bone formation and bone healing. A recent literature review found that most in vivo models with positive results used large bone defects in dog’s tibia with DC (direct current) treatment to prove the effects of ES on bone healing and that most clinical trials are made on tibia’s delayed/non-unions fracture using pulsed electromagnetic field (PEMF) showing positive results [72]. At the same time, they asked orthopedic surgeons through a survey about the use of ES to treat bone fracture and despite the awareness and positive impression only around 30% of the responders claimed to have used it and 85% said that they would be willing to use this technology if an easy-to-use device was available to treat bone fracture [72]. The lack of well-evidenced protocols and high cost were some of the problems the orthopedic surgeons determined, very similar to the drawbacks that the SLP reported in the survey reviewed previously. This is why is important to mention that clinicians and researchers are making the effort to counter these issues in the different fields ES is used as a recent study determined the dose-response relationship between NMES and muscle function in patients with rheumatoid arthritis using a curve estimation regression statistics concluding that the minimum NMES training intensity necessary to obtain significant results is around 15% of the maximum voluntary contraction [73], this represents one step closer to high quality evidence-based practice for NMES treatments. Apart from that, it was noticed that few studies intent to show the effects of intraoral electrical stimulation for OPD but mostly for treating snoring. Wessolleck et al. [74] tries a newly developed intraoral stimulator to improve snoring in patient with Obstructive Sleep Apnea (OSA) under the understanding that oropharyngeal exercises and electrostimulation may increase muscle tone above the base tone during sleep preventing the collapse of the pharynx resulting in snoring. In this study ES was applied twice a day for 20 min during the 6-week treatment resulting in a statistically significantly reduce of snoring which was lowered on average by 44% after treatment and remained stable for 2 weeks post-treatment (43%). The individuals with Apnea-Hypopnoea Index (AHI) below 10 benefited the most, decreasing the snoring score on 68% average. The authors claim no undesirable effects arose in the study [74]. Another scope of this ES method has reach neural regeneration as a promoting effect of neurite outgrowth [75]. It has been demonstrated that a single session of 1 h of electrical stimulation is sufficient to enhance axon regeneration in hindlimb common peronea nerve after repairing the nerve itself and when compared to sham ES significantly more motor and sensory neurons were regenerated [76].

These are examples of scientific research that are leaving the door open for investigating the effect of a safe and localized NMES treatment, opportunity that we are not missing as we decided to investigate about this matter and develop the idea of combining the ES effects on hard and soft tissue regeneration and its possible intraoral use to solve dental problems and also setting the basis for further therapeutic effects of electrical stimulation.

## Perspectives and Closing Remarks

In general, ES has thus far, come a long way in different disciplines of Medicine. Whether to train and re-habilitate muscle function or to enhance bone healing, such technology has and continues to be promising as well as expanding its potential applications. One such recent novel area for ES is neuroscience, where ES can be employed to manipulate what the brain controls including the motor and cognitive pathways. Indeed, it was demonstrated how trans-cranial direct current stimulation (applied to the left dorso-lateral pre-frontal cortex) was able to modulate cortical activity in younger and older healthy adults, in varying ways, and depending on factors such as age and the presence/absence of mild cognitive impairment [77]. Further, ES for deep brain stimulation (DBS) on the sub-thalamic nucleus of patients suffering from Parkinson’s disease resulted in the successful reduction of tremor, muscle stiffness and bradykinesia (slowness of movement), thereby boosting the research on the applicability and potentially-therapeutic effects of DBS in treating other pathologies [78]. Indeed, a proof-of-concept pre-clinical study (in a rat model) demonstrated the effects of DBS on modulating noise-induced tinnitus, a condition known to diminish QoL of patients, with lasting effects of up to 5 days [78]. Furthermore, an “ingestible” electrical device designed to stimulate the gastro-intestinal tract tissues has been unveiled and is intended for use in tackling obesity and potentially-replacing by-pass gastric surgeries as it induces satiety [79, 80]. Last but not least, it might be worth mentioning that in our BioMAT’X R&D&I Laboratory, we are also currently employing NMES principles in the design and evaluation of a prototype device that can be utilized in locally enhancing tissue regeneration and repair. Such ES-based advances and technological prospects demonstrate the potential of NMES in rehabilitation and QoL improvement for patients suffering from a wide range of conditions.

## Conclusions

Regardless of the accruing evidence supporting NMES, it is clear that more high-quality studies with larger numbers of patients are required to better identify the pros and cons of electrical therapy in the rehabilitation of OPD and TMD, amongst others. We need to better comprehend the underlying mechanisms and how muscles respond and react to electrical current(s), bio-mechanically and physiologically. Such research should also address the effectiveness, efficacy and safety, along with the potentially-influencing factors, including optimal dosage, stimulation and delivery parameters, timing and protocols. It is hence,vital important for any clinician caring for a dysphagia patient to be knowledgeable and up-to-date regarding dysphagia management, in order to provide the safest and most effective and efficacious treatment modalities and alternatives, thereby to improve QoL, shorten hospitalization time, recover the oral route and prevent the complications of dysphagia. Such commitment also applies to all other NMES indications, including TMD therapy and dentists.

## Data Availability

Not Applicable.
